# Multi-task deep learning integrating pretreatment MRI and whole slide images predicts induction chemotherapy response and survival in locally advanced nasopharyngeal carcinoma

**DOI:** 10.1371/journal.pdig.0001378

**Published:** 2026-10-05

**Authors:** Jing Hou, Xiaochun Yi, Congrui Li, Junjian Li, Hui Cao, Qiang Lu, Xiaoping Yu

**Affiliations:** 1 Department of Diagnostic Radiology, Hunan Cancer Hospital and the Affiliated Cancer Hospital of Xiangya School of Medicine, Central South University, Changsha, Hunan, People's Republic of China; 2 Hunan Provincial Key Laboratory on Bioinformatics, School of Computer Science and Engineering, Central South University, Changsha, People's Republic of China; 3 Department of Health Service Center, Hunan Cancer Hospital and the Affiliated Cancer Hospital of Xiangya School of Medicine, Central South University, Changsha, Hunan, People's Republic of China; Tianjin Medical University General Hospital, CHINA

## Abstract

Predicting response to induction chemotherapy (IC) and overall survival (OS) is critical for optimizing treatment in patients with locally advanced nasopharyngeal carcinoma (LANPC). This study aimed to develop and validate a multi-task deep learning model integrating pretreatment MRI and whole slide images (WSIs) to predict IC response and OS in LANPC. Pretreatment MRI and WSIs from 404 patients with LANPC were retrospectively collected to construct a multi-task model (MoEMIL) for the simultaneous prediction of early IC response and OS. In our cohort, no progressive disease cases were observed; therefore, the poor responder group consisted exclusively of patients with stable disease. MoEMIL employed multi-instance learning to process WSIs, PyRadiomics and a convolutional neural network (ResNet50) to extract MRI features, and fused multimodal features through a multi-gate mixture-of-experts architecture. Clustering-constrained attention multiple instance learning and gradient-weighted class activation mapping were applied for visualization and interpretation. MoEMIL effectively stratified patients into good and poor IC response groups, achieving areas under the curve of 0.917, 0.869, and 0.801 in the train, validation, and test sets, respectively. In the test set, MoEMIL significantly outperformed the deep learning radiomics model and TNM staging for both endpoints. Compared to the Pathomics model, MoEMIL achieved significant improvement for OS (*P* = 0.0049) and borderline improvement for IC response (*P* = 0.064). The model could stratify patients into high- and low-risk OS groups (*P <* 0.05). MoEMIL might be an adjunctive tool for early induction chemotherapy response prediction and prognostic stratification in LANPC.

## Author summary

We have developed a deep learning model that integrates two types of medical images, including magnetic resonance imaging (MRI) and digital pathological slices, to simultaneously predict response to induction chemotherapy and prognosis in patients with locally advanced nasopharyngeal carcinoma. Current treatment decisions primarily rely on traditional tumor staging (TNM), which often fails to comprehensively reflect the complexity of the disease. Our model, named MoEMIL, was trained and tested on data from 404 patients across two hospitals and consistently outperformed both single-model approaches and TNM staging methods. By identifying patients who exhibit poor response to induction chemotherapy or higher prognostic risk, our tool may assist clinicians in risk stratification and treatment planning, enabling intensified management for high-risk patients and avoiding unnecessary side effects for low-risk patients, but prospective validation is required before routine clinical adoption. Additionally, we visualize the model’s reasoning process through heat map generation, which highlights the image regions exerting the greatest influence on prediction outcomes. This work represents a step toward more precise treatment for nasopharyngeal carcinoma; however, larger-scale prospective studies are required before the model can be integrated into routine clinical practice.

## Introduction

Nasopharyngeal carcinoma (NPC) is an epithelial malignancy with a distinct geographical prevalence in East and Southeast Asia [1,2]. Approximately 70–80% of patients are diagnosed with locally advanced nasopharyngeal carcinoma (LANPC), which is generally classified as stage III or IVa [1]. The current standard treatment for LANPC involves induction chemotherapy (IC) followed by concurrent chemoradiotherapy (CCRT) [3], which has been shown to improve survival outcomes compared to CCRT alone. However, not all patients respond well to IC. Moreover, approximately 20%-30% of LANPC patients ultimately experience treatment failure [4]. Local recurrence and distant metastasis are the main reasons for treatment failure. Tumor response to IC is an independent prognostic factor for survival after intensity-modulated radiation therapy in NPC [5]. Accurate risk assessment following IC could guide timely adjustments to subsequent CCRT, potentially improving survival while reducing unnecessary treatment-related toxicity.

The Tumor-Node-Metastasis (TNM) staging system remains the primary method for guiding therapeutic decisions and prognostication in patients with LANPC [6]. However, its reliance on anatomical details overlooks the inherent heterogeneity within tumors, often leading clinicians to observe disparities in early treatment response and overall prognosis among patients staged identically according to TNM. This limitation underscores the urgent need for more refined approaches that can capture the complex biological characteristics of tumors.

Medical imaging provides a noninvasive way to monitor tumor response and progression during treatment. Advances in radiomics enable the transformation of visual medical images into mineable high-dimensional data through high-throughput computing, a technique that has been widely applied to predict tumor response and survival outcomes in the early stage of LANPC [7]. Compared with conventional radiomics, deep learning automatically quantifies complex tumor characteristics beyond human visual perception and has attracted considerable attention in medical imaging research due to its superior performance. Deep survival models have demonstrated the potential to outperform conventional radiomics-based prognostic prediction models [8–12]. However, most current models for IC prediction and prognosis focused on performing a single task method [13–15], unable to capture inter-task relationships or effectively model the complex nonlinear interactions between various prognostic factors. In contrast, multi-task learning allows multiple related tasks to be learned simultaneously within a single model. By sharing feature representations between related tasks, multitask learning is more data-efficient and has been shown to reduce overfitting and improve model generalizability in many tumors [16], including gastric cancer [17,18], lung cancer [19].

In addition to radiological imaging, pathological examination plays a crucial role in diagnosing NPC, directing clinical treatment, and forecasting prognosis. The former offers macroscopic details about the tumor, whereas the whole-slide images (WSIs) of the latter can thoroughly disclose the biological traits and microenvironment of the tumor. The integration of radiological and pathological data, known as radiopathomics, has shown promise in enhancing predictive accuracy by capturing tumor information at different scales. This approach has been successfully applied to prognosis prediction and therapeutic efficacy assessment in various solid tumors, including breast cancer [20], prostate cancer [21], and rectal cancer [22]. However, its application in NPC remains limited, with few studies leveraging the complementary information provided by these multimodal data sources, especially in the multi-task prediction.

In light of these considerations, we aimed to develop and validate an integrative radiopathomics model based on deep learning for simultaneously predicting response to IC and clinical outcomes in patients with LANPC. The model employs multi-instance learning to process WSIs, a convolutional neural network (ResNet50) to extract MRI features, and integrates these multimodal features through a multi-gate mixture of experts (MMoE) architecture [23]. By leveraging multi-center data and explainable artificial intelligence techniques, we sought to create a robust, interpretable predictive tool that can ultimately support personalized treatment planning for LANPC patients, addressing the limitations of current unimodal and single-task approaches.

Although we previously developed a deep learning radiopathomics model for predicting overall survival (OS) in LANPC using a partially overlapping cohort [24], the present study differs fundamentally by introducing a multi-task framework that simultaneously predicts both IC response and OS, and by employing a novel MMoE architecture for adaptive multimodal feature fusion. The main aims of this study were (1) the development of a novel multi-task deep learning framework that simultaneously predicts IC response and OS in patients with LANPC; (2) the integration of MRI and WSI data to capture complementary information at different scales; and (3) comprehensive validation using multi-center data and demonstration of clinical utility through survival stratification.

## Materials and methods

### Patients

This retrospective study was approved by the institutional review board, and the requirement for written informed consent was waived. A total of 404 eligible LANPC patients were included in this study. The primary cohort consisted of 364 patients retrospectively enrolled from Hunan Cancer Hospital (January 2014 to June 2018). An external test cohort of 40 patients was recruited from the First Affiliated Hospital of Jinan University (Guangzhou, China). As shown in Table A in S1 Text, compared to our previous study [24], the current study added 71 new patients from Hunan Cancer Hospital (enrolled between June 2018 and December 2019) and excluded 10 patients from the external cohort due to incomplete IC response assessment. Inclusion criteria were: (1) age ≥ 18 years; (2) histologically confirmed NPC, classified as stage III to IVa according to the 7th before 2017 or 8th after 2017 edition of the AJCC TNM staging system; (3) received IC followed by CCRT; (4) availability of complete pretreatment contrast-enhanced T1-weighted (CET1-w) MRI sequences; and (5) availability of high-quality hematoxylin and eosin (H&E)-stained biopsy slides obtained via nasopharyngoscopy. Exclusion criteria were: (1) history of other malignancies or prior anticancer therapies; (2) poor-quality H&E-stained slides (e.g., excessive staining artifacts); and (3) incomplete efficacy assessment data after IC and follow-up data. The patient selection process is detailed in Fig 1.

**Fig 1:**
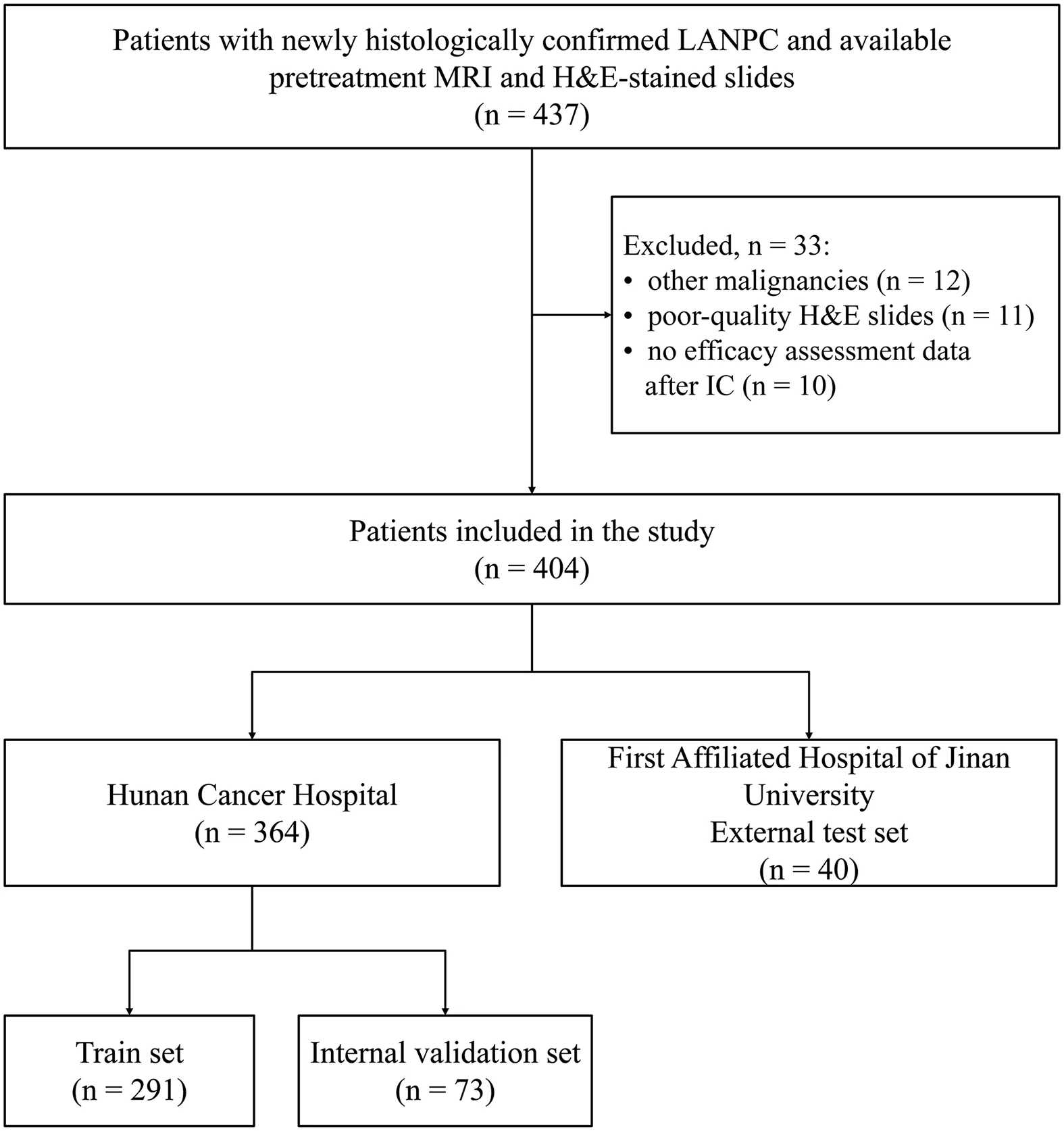
Flow-chart shows patient recruitment process. Abbreviations: LANPC = locally advanced nasopharyngeal carcinoma, MRI = magnetic resonance imaging, IC = induction chemotherapy.

IC regimens were cisplatin-based, administered every 3 weeks for 2–4 cycles. Radiotherapy was delivered with a total dose of 70–76 Gy in 30–33 fractions. Concurrent chemotherapy during radiotherapy involved cisplatin for 3–6 cycles. Follow-up was conducted per National Comprehensive Cancer Network (NCCN) guidelines [3]. Nasopharyngeal MRI follow-up evaluations were performed every 3 months in the first year, every 6 months in the second year, and annually thereafter. The curative response to IC was classified into complete response (CR; with no evidence of residual disease on MRI and nasopharyngoscopy, regression ratio of 100%), partial response (PR; with evidence of residual disease, regression ratio >30%), stable disease (SD; with evidence of residual disease, regression ratio <30%) and progressive disease (PD; with evidence of increase, increase ratio>20%) according to the Response Evaluation Criteria in Solid Tumors (RECIST) 1.1 guidelines [25]. CR and PR are considered as good responders, while SD and PD are classified as poor responders. The primary endpoint was OS, defined as the time from diagnosis to death from any cause.

### MRI acquisition and segmentation

MRI scans were conducted at both hospitals using 1.5-Tesla MRI scanners with combined head and neck coil. At our hospital, the imaging sequence parameters were: Axial T1‑weighted spin‑echo: repetition time (TR)/echo time (TE) = 580/7.8 ms, slice thickness = 5 mm, 36 slices, interslice spacing = 1 mm, and number of excitations (NEX) = 2. Axial fat‑suppressed T2‑weighted spin‑echo: TR/TE = 6289/85 ms, slice thickness = 5 mm, 36 slices, interslice spacing = 1 mm, NEX = 2. Axial contrast‑enhanced T1‑weighted spin‑echo (CET1‑w): TR/TE = 500/8 ms, field of view (FOV) = 22 × 22 cm, NEX = 2, slice thickness = 4 mm, interslice spacing = 0.8 mm. For the CET1‑w images, a gadolinium‑based contrast agent (gadodiamide) was injected intravenously at a dose of 0.1 mmol/kg body weight, with a flow rate of 3.5 mL/s. At the First Affiliated Hospital of Jinan University, the imaging sequence parameters were: Axial T1‑weighted imaging (T1WI): TR/TE = 500/8 ms, slice thickness = 4 mm, interslice spacing = 1 mm, NEX = 2. Axial T2‑weighted imaging (T2WI): TR/TE = 5000/85 ms, slice thickness = 4 mm, interslice spacing = 1 mm, NEX = 2. Axial CET1‑w: TR/TE = 500/8 ms, FOV = 22 × 22 cm, slice thickness = 4 mm, interslice spacing = 1 mm, NEX = 2.

MRI preprocessing was conducted using AK software (Artificial Intelligence Kit, GE Healthcare), which has been officially registered and approved. The preprocessing involved resampling, skull stripping, and intensity normalization. The image resolution was adjusted to 1 × 1 × 1 mm during resampling, with all MRI image thicknesses standardized to 1 mm through linear interpolation. A gray-level adjustment was then applied to standardize the intensity range to 0–255. Two experienced radiologists (A with 16 and B with 15 years of experience in head and neck MRI, respectively), blinded to clinical outcomes, independently delineated the primary NPC lesions layer by layer using ITK-SNAP software (version 3.8.0; http://www.itksnap.org/). Inter- and intraclass correlation coefficients (ICCs) were employed to assess segmentation reproducibility. Radiologists A and B independently segmented MRI scans from 30 randomly selected LANPC patients, and Radiologist A repeated the segmentation one month later to assess intra-reader consistency. The intra-reader ICC for Radiologist A ranged from 0.751 to 0.997, while the inter-reader ICC between the two radiologists ranged from 0.794 to 0.990. All ICCs were consistently above 0.75, indicating excellent reproducibility. Based on these findings, Radiologist A was assigned to segment the remaining images.

### Radiomics features and deep learning features extraction

To capture comprehensive tumor phenotypic information, we designed a dual-branch feature extraction framework that integrates traditional radiomics features with deep learning-derived features. Radiomics features were extracted using the IBSI-compliant PyRadiomics toolkit to quantify tumor texture, shape, and intensity characteristics [26]. A total of 107 features were computed, including 14 morphological descriptors, 18 first-order statistical features, and 75 second-order texture features derived from gray-level co-occurrence matrices (GLCM), gray-level run-length matrices (GLRLM), gray-level size zone matrices (GLSZM), neighborhood gray-level difference matrices (NGTDM), and gray-level dependence matrices (GLDM). To construct a unified feature set, we treated the distinct radiomics categories (morphology, statistics, texture) and the deep features as radiology-based instances and fed them into instance-wise fully connected layers, respectively, to generate radiology embeddings *R*_*bag*_ = {r_l_}∈R^L×dr^. In parallel, the same VOIs were fed into a pretrained ResNet-50 convolutional neural network (CNN) with its final classification layer removed to extract deep learning features. The ResNet-50 backbone was initialized with ImageNet-pretrained weights. Given the small batch size of 1 during training, all BatchNorm layers in the pretrained backbone were kept in evaluation mode, and their running mean and variance were not updated during fine-tuning. This was done to avoid unstable BatchNorm statistics caused by the very small batch size. The affine parameters of BatchNorm layers were also frozen. The resulting deep features were also transformed through a separate linear layer, to generate CNN embeddings *R*_*deep*_∈R^l×dr^. Finally, the radiomics features and deep CNN features were fused using an attention mechanism to form a unified radiological feature representation for downstream analysis.

### Histological features encoding from WSIs

Histological feature extraction was performed using a deep learning-based, weakly supervised framework adapted from Clustering-constrained Attention Multiple Instance Learning (CLAM) [27]. H&E-stained tissue sections were digitally scanned at 40 × magnification using a Pannoramic MIDI II scanner, yielding WSIs in SVS format with a resolution of 0.14 µm/pixel. Following the CLAM preprocessing pipeline, Otsu’s thresholding was first applied to separate foreground tissue from the background. Given the gigapixel size of WSIs, which prevents direct network input, the tissue regions were segmented into non-overlapping patches of 256 × 256 pixels. Subsequently, each patch was encoded using the pre-trained CONCHv1.5 visual encoder, which serves as the patch-level feature extractor of the TITAN foundation model, to obtain high-quality latent representations [28]. To aggregate these patch features into a single slide-level representation, an attention-based Multiple Instance Learning framework was employed [29,30]. In this approach, all patches from a WSI are treated as a “bag” of instances. The network learns attention weights that reflect the diagnostic relevance of each patch, and this is followed by a weighted summation of the patch features. This process allows the model to focus on morphologically informative regions while suppressing contributions from non-tissue background, ultimately generating a compact, global feature vector for the entire WSI. Furthermore, attention heatmaps were generated based on the attention scores derived from the pooling layer to visualize the importance of each region within the WSI.

### Multimodal features fusion via MMoE

Before the MMoE module, the above-extracted radiological and pathological feature vectors were initially fused and concatenated to form a combined multimodal representation. Subsequently, this combined feature vector was sent to a fusion module, which consists of a linear layer for dimensionality reduction and feature mapping, followed by the ReLU activation function and dropout regularization to introduce nonlinearity and reduce overfitting. This step not only standardizes the feature dimensions but also prepares the fused representation for processing in the subsequent MMoE module.

The MMoE module serves as the core innovative component of the model, designed to enable effective multi-task feature sharing. This process consists of three parts, namely the expert network, the task-specific gating network, and the weighted feature fusion. Expert Network: This module utilizes multiple feed-forward neural networks (i.e., experts), which are structurally identical but have independent parameters. During the implementation process, each expert is implemented as a two-layer multi-layer perceptron (MLP), with a structure of linear → ReLU → dropout → linear → ReLU. All specific task features from the previous fusion stage are processed in parallel through these expert networks, thereby generating a set of diverse feature representations. Gating Network: Two independent gating networks are respectively set up for two different downstream tasks, namely IC response evaluation and survival prediction. Each gating network acts as a lightweight attention mechanism, which receives input features and outputs a normalized weight vector (for example, [0.2, 0.1, 0.7]), reflecting the relevance of each expert’s output to the current task. Weighted Feature Fusion: For each specific task, the weighted vector calculated by the corresponding gating network is multiplied to the outputs of each expert network and then summed. Through this dynamic weighting mechanism, each task ultimately obtains a feature representation tailored to it, which is composed of different expert features according to the task requirements, thereby effectively alleviating the problem of negative transfer.

### MoEMIL model construction and validation

Following the MMoE module, this component generates adaptive feature representations tailored to the specific task. These features are then routed to distinct prediction heads to produce the final clinical output. The IC response evaluation head receives the first task-specific feature vector from the MMoE output and processes it through a MLP. The MLP output corresponds to classification prediction values for different response categories. The survival prediction head processes the second task-specific feature vector via another MLP to generate a risk score. This score can be utilized to generate Kaplan-Meier survival curves, thereby facilitating the assessment of patient prognosis.

The proposed multi-task model was implemented in PyTorch (version 2.1.2) and trained on a single NVIDIA GeForce RTX 3080 Ti GPU. During training, we used a batch size of 1 and the Adam optimizer with an initial learning rate of 2 × 10^−4^. A dropout rate of 0.3 was applied after the task-specific towers to reduce overfitting. The MMoE backbone was configured with 3 experts. The model was trained for a maximum of 100 epochs with early stopping patience of 8 epochs based on the validation loss. The total loss function was defined as:


$$\begin{array}{c} L_{total}=L_{IC}+L_{OS} \end{array}$$

where *L*_*IC*_ is the binary cross-entropy loss for IC response classification (responder vs. non-responder), and *L*_*OS*_ is the discrete-time survival negative log-likelihood loss for OS. The loss function used in this study follows the cross-entropy-based discrete-time survival formulation described by Zadeh and Schmid (IEEE TPAMI, 2020) [31]. For *L*_*OS*_, we partitioned the follow-up time into four intervals based on the quartiles of the survival distribution. For uncensored patients, the loss was computed using the interval in which the event occurred. For censored patients, the likelihood contribution was defined based on survival beyond the last observed follow-up interval, so that censored cases contributed information up to their censoring time without being treated as events. No additional weighting was applied between the two loss terms. This equal-weight strategy was adopted because both components are negative log-likelihood-type objectives and their magnitudes were empirically comparable during training.

The predictive and prognostic performances of the Deep Learning Radiomics (DLR), Pathomics, and integrated model named Mixture-of-Experts integrated Multiple Instance Learning (MoEMIL) models were quantitatively evaluated using the receiver operating characteristic (ROC) curve and concordance index (C-index). Additionally, Kaplan-Meier curve analysis was conducted to demonstrate the models’ ability to stratify LANPC patients into high- and low-risk groups. High- and low-risk groups were defined using the median predicted risk score derived from the training cohort, and the same predefined risk threshold was applied to the validation and external test cohorts. The workflow of the study is depicted in Fig 2.

**Fig 2:**
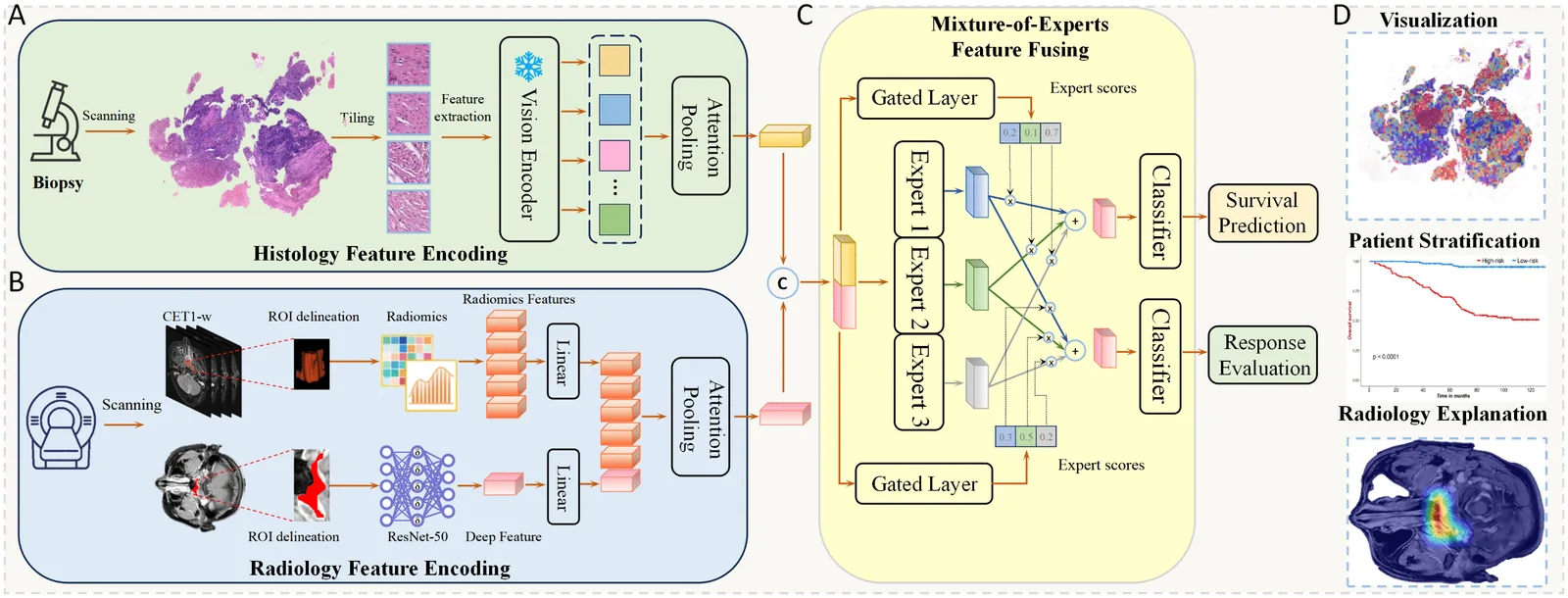
Workflow of this study. The workflow comprises four main steps, and the model architecture consists of three core components, including the feature encoding module (MRI and WSI feature encoding), the multimodal fusion module, and the task-specific output heads. **(A)** Histology feature encoding: WSIs are processed by first cropping them into clinical-grade patches of 256 × 256 pixels at 20 × magnification. These patches are then fed into the CONCHv1.5 visual encoder (from the TITAN foundation model) to extract high-dimensional latent representations. An attention pooling mechanism is subsequently employed to aggregate these patch-level features into a global histological feature vector, effectively identifying the most diagnostically relevant regions. **(B)** Radiology feature encoding: This module extracts complementary information from multi-parametric MRI (including CET1-w sequences). It combines hand-crafted radiomic features (capturing texture and shape) with deep CNN features (extracted via a ResNet-50 backbone). These heterogeneous radiological signatures are fused through an attention-based bottleneck to generate a unified radiology feature representation. **(C)** Mixture-of-experts feature fusion: This process utilizes an MMoE architecture. The fused multimodal input is processed by a set of shared experts, while dedicated gated layers learn to assign specific weights (expert scores) to each expert’s output. This allows the model to dynamically balance the contribution of histological and radiological data based on the specific requirements of each downstream task. **(D)** Model visualization and clinical application: For WSIs, attention heatmaps highlight morphological regions contributing to high-risk scores. For MRI, Grad-CAM generates saliency maps to visualize the volumetric basis of the model’s decision-making. Abbreviations: CET1-w = contrast-enhanced T1-weighted spin-echo image, WSI = whole slide image, MIL = multiple instance learning, Grad-CAM = Gradient-weighted Class Activation Mapping.

### Model interpretation and visualization

To interpret the importance of different tissue regions for prognostic predictions in a WSI, attention scores were computed for all overlapping 256 × 256 tissue patches within a WSI. These scores were normalized to a percentile scale from 0.0 (low attention) to 1.0 (high attention) and spatially registered back to the original slide. For visualization, the scores were converted into an RGB heatmap using a color map and overlaid on the corresponding H&E-stained image at 50% transparency. Regions with high attention (typically colored red) frequently corresponded to morphologically distinct tumor areas, whereas regions with lower attention (often blue) included normal tissue and background. This method provides an intuitive, annotation-free visualization of the tissue regions that the model deems most relevant for prognosis.

For interpretation and visualization of MRI, gradient-weighted class activation mapping (Grad- CAM) algorithm was used to identify and visualize the regions of the MRIs that play a crucial role in the model’s predictions [32]. This method provides explainability by visualizing, in the form of a heatmap, the area on which the model focuses when making predictions, intuitively showing the basis used by the model to make decisions. The code is publicly available at https://github.com/junjianli106/PathRadMoE.

### Statistical analysis

Quantitative data were presented as mean (standard deviation [SD]) or median (interquartile range [IQR]). Continuous variables were compared using Student’s t test or Mann-Whitney U test, and categorical variables were compared using the χ² test or Fisher’s exact test. All statistical analyses were two-sided, and *P* values of less than 0.05 indicated statistical significance. Area under the curve (AUC) of the ROC curve was applied to assess the accuracy of the DLR, pathomics, and MoEMIL models for predicting IC response. DeLong tests for AUC comparisons and paired bootstrap (1000 resamples) for C-index comparisons were performed to compare MoEMIL against each unimodal model in the test set. Kaplan-Meier curve and log-rank tests were applied to assess survival outcomes among subgroups. The risk threshold used for Kaplan-Meier stratification was defined a priori based on the training cohort only. Concordance index(C-index) was used to assess the prognostic performance of the DLR, pathomics, and MoEMIL models. Furthermore, a subgroup analysis was performed to evaluate whether EBV-DNA provides complementary value to the MoEMIL model in patients with available EBV-DNA data. All statistical analyses were performed using R version 4.3.0 (2024-02-29), along with Storm Statistical Platform (www.medsta.cn/software), or Python (version 3.8.18) with the scikit-learn package (version 1.3.2), PyTorch (version 2.1.2), NumPy (version 1.24.1), Pandas (version 2.0.3), and OpenSlide (version 1.3.1).

## Results

### Patient characteristics

The initial sample group of this study consisted of 437 patients with LANPC. Among them, 33 patients were excluded due to having other malignant tumors (n = 12), poor quality of H&E-stained slides (n = 11), and no efficacy assessment data after IC (n = 10). Finally, 404 LANPC patients were included in the study (265 men, 139 women), with a mean age of 46.59 ± 9.54 years. The number of patients per category was: CR = 2, PR = 315, SD = 87. Good responders (CR + PR) totaled 317. Because PD was absent, the poor responder group consisted exclusively of SD (n = 87). These patients were allocated into a train set (n = 291), a validation set (n = 73), and an external test set (n = 40). The median follow-up duration was 87.63 months (interquartile range, IQR: 53.00-109.03). Across the three cohorts, the number of OS events (deaths) and censored observations were as follows: in the training set, there were 45 deaths and 246 censored cases; in the validation set, there were 11 deaths and 62 censored cases; and in the external test set, there were 5 deaths and 35 censored cases. Baseline clinical characteristics, including age, sex, T stage, N stage, TNM stage, and differentiation degree, showed no significant differences across the three cohorts (Table 1), which confirmed a well-balanced data partition.

**Table 1:** Characteristics of patients in the train, validation, and test sets.

Variables	Total (n = 404)	Train set (n = 291)	Validation set (n = 73)	Test set (n = 40)	Statistic	*P*
age, Mean ± SD	46.59 ± 9.54	46.63 ± 9.12	45.03 ± 9.55	49.17 ± 11.95	F = 2.47	0.086
Sex, n (%)					χ^2^ = 4.63	0.099
Female	139 (34.41)	99 (34.02)	31 (42.47)	9 (22.50)		
Male	265 (65.59)	192 (65.98)	42 (57.53)	31 (77.50)		
T stage, n(%)					χ^2^ = 8.58	0.199
T 1	32 (7.92)	26 (8.93)	4 (5.48)	2 (5.00)		
T 2	159 (39.36)	120 (41.24)	27 (36.99)	12 (30.00)		
T 3	138 (34.16)	90 (30.93)	27 (36.99)	21 (52.50)		
T 4	75 (18.56)	55 (18.90)	15 (20.55)	5 (12.50)		
N stage, n(%)					–	0.086
N0	15 (3.71)	8 (2.75)	3 (4.11)	4 (10.00)		
N1	38 (9.41)	28 (9.62)	3 (4.11)	7 (17.50)		
N2	255 (63.12)	185 (63.57)	49 (67.12)	21 (52.50)		
N3	96 (23.76)	70 (24.05)	18 (24.66)	8 (20.00)		
TNM, n(%)					χ^2^ = 2.23	0.327
III	247 (61.14)	183 (62.89)	39 (53.42)	25 (62.50)		
IVa	157 (38.86)	108 (37.11)	34 (46.58)	15 (37.50)		
Differentiation degree, n(%)					–	0.083
WHO type I	1 (0.25)	1 (0.34)	0 (0.00)	0 (0.00)		
WHO type II	89 (22.03)	71 (24.40)	15 (20.55)	3 (7.50)		
WHO type III	314 (77.72)	219 (75.26)	58 (79.45)	37 (92.50)		
EBV-DNA (copies/mL), n(%)					χ^2^ = 4.53	0.339
≤ 4 × 10^2^	112 (27.72)	89 (30.58)	16 (21.92)	7 (17.50)		
> 4 × 10^2^	105 (25.99)	73 (18.07)	20 (27.40)	12 (30.00)		
Missing	187 (46.29)	129 (44.33)	37 (50.68)	21 (52.50)		

F: F-test, χ^2^: Chi-square test, -: Fisher exact.

SD: standard deviation.

Abbreviations: WHO = world health organization, EBV-DNA = Epstein-Barr Virus DNA.

### Prediction of induction chemotherapy response

The predictive performance for IC treatment response is summarized in Table 2. TNM staging showed poor performance, with AUCs of 0.713 (95% CI: 0.620-0.806), 0.682 (95% CI: 0.596-0.768), and 0.645 (95% CI: 0.533-0.767) in the train, validation, and test sets, respectively. The unimodal DLR and Pathomics models demonstrated moderate performance. Specifically, the DLR model achieved AUCs of 0.774 (95% CI: 0.682-0.867), 0.711 (95% CI: 0.624-0.797), and 0.709 (95% CI: 0.587-0.831) in the train, validation, and test sets, respectively. The Pathomics model yielded AUCs of 0.832 (95% CI: 0.745-0.918), 0.772 (95% CI: 0.689-0.858), and 0.752 (95% CI: 0.680-0.824) across the same sets. In contrast, the integrated MoEMIL model attained the highest AUC in all datasets (Fig 3): 0.917 (95% CI: 0.848-0.986), 0.869 (95% CI: 0.790-0.947), and 0.801 (95% CI: 0.726-0.876) in the train, validation, and test sets, respectively, demonstrating a 19.5% improvement over the TNM staging, 11.5% over DLR model, and a 6.1% marginal improvement over the Pathomics model. In the external test set, DeLong test demonstrated MoEMIL significantly outperformed the DLR model (*P* = 0.015) and TNM staging (*P* < 0.001). Compared to the Pathomics model, MoEMIL achieved borderline improvement for IC response (*P* = 0.064) (Table A in S1 Text). Calibration plots and decision curve analysis of models were shown in S1 Fig.

**Table 2:** Predictive performance of three models in LANPC patients.

Models	Train set		Validation set		Test set	
	AUC	ACC	AUC	ACC	AUC	ACC
TNM	0.713 (0.620,0.806)	0.790 (0.694,0.886)	0.682 (0.596,0.768)	0.714 (0.627,0.801)	0.645 (0.533,0.767)	0.700 (0.596,0.814)
DLR	0.774 (0.682,0.867)	0.769 (0.672,0.865)	0.711 (0.624,0.797)	0.718 (0.631,0.805)	0.709 (0.587,0.831)	0.752 (0.638,0.866)
Pathomics	0.832 (0.745,0.918)	0.823 (0.734,0.913)	0.772 (0.686,0.858)	0.740 (0.652,0.829)	0.752 (0.680,0.824)	0.786 (0.688,0.884)
MoEMIL	0.917 (0.848,0.986)	0.858 (0.771,0.946)	0.869 (0.790,0.947)	0.803 (0.715,0.892)	0.801 (0.726,0.876)	0.797 (0.715,0.879)

Abbreviation: LANPC = locally advanced nasopharyngeal carcinoma, DLR = deep learning radiomics, MoEMIL = Mixture-of-Experts integrated Multiple Instance Learning.

**Fig 3:**
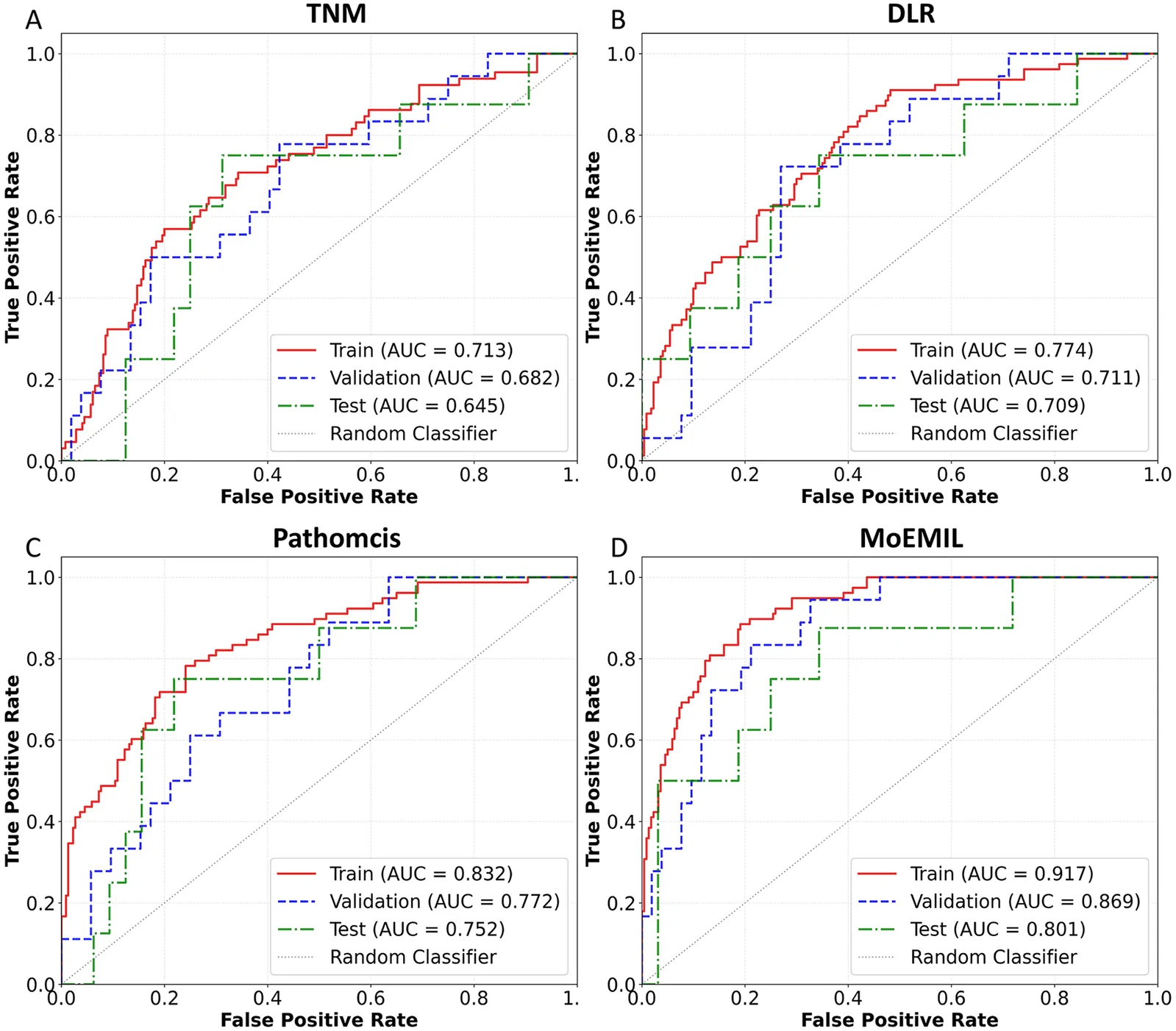
Predictive analysis for predicting IC response in LANPC patients using TNM staging, DLR, Pathomics, and MoEMIL models across the train, validation, and external test sets. Abbreviations: IC = induction chemotherapy, LANPC = locally advanced nasopharyngeal carcinoma, DLR = deep learning radiomics, MoEMIL = Mixture-of-Experts integrated Multiple Instance Learning.

### Prognostic performance for overall survival

The predictive performance for survival is summarized in Table 3. The TNM staging and DLR models showed relatively lower OS prognostic accuracy, with C-indices of 0.689 (95% CI: 0.639-0.739) and 0.657 (95% CI: 0.605-0.710) in the train set, respectively. In contrast to DLR model, TNM staging demonstrated lower prognostic efficacy in both the validation and test sets (0.587 and 0.553 for TNM staging vs. 0.655 and 0.676 for the DLR model). In comparison, the Pathomics model exhibited improved performance, yielding C-indices of 0.765 (95% CI: 0.712-0.820), 0.681 (95% CI: 0.602-0.805), and 0.690 (95% CI: 0.650-0.701) across the same sets. The MoEMIL model outperformed both unimodal approaches and TNM staging, achieving the highest predictive accuracy across all cohorts: a C-index of 0.785 (95% CI: 0.701-0.854) in the train set, 0.773 (95% CI: 0.655-0.835) in the validation set, and 0.766 (95% CI: 0.701-0.853) in the test set. Based on the test set C-indices the MoEMIL model demonstrated a 27.8% improvement over the TNM staging and 11.7% over DLR model, and a 9.9% improvement over the Pathomics model. In the external test set, paired bootstrap demonstrated MoEMIL significantly outperformed the DLR model (*P* < 0.001), TNM staging (*P* < 0.001), and Pathomics (*P* = 0.0049) model for OS (Table B in S1 Text).

**Table 3:** Prognostic performance of three models in LANPC patients.

Models	Train set		Validation set		Test set	
	C-index (95%CI)	*P*	C-index (95%CI)	*P*	C-index (95%CI)	*P*
TNM	0.689 (0.639,0.739)	0.012	0.587 (0.537,0.637)	0.014	0.553 (0.463,0.643)	0.041
DLR	0.657 (0.605,0.710)	<0.001	0.655 (0.603,0.707)	<0.001	0.676 (0.564,0.767)	<0.001
Pathomics	0.765 (0.712,0.820)	<0.001	0.681 (0.602,0.805)	0.009	0.690 (0.650,0.701)	<0.001
MoEMIL	0.785 (0.701,0.854)	0.001	0.773 (0.655,0.835)	<0.001	0.766 (0.701,0.853)	<0.001

Abbreviation: LANPC = locally advanced nasopharyngeal carcinoma, DLR = deep learning radiomics, MoEMIL = Mixture-of-Experts integrated Multiple Instance Learning.

To quantify the stratification performance of the risk groups, we calculated the hazard ratios (HRs) for high-risk versus low-risk patients. The HRs were 13.51 (95% Cl 6.22-29.35, *P* < 0.0001) in the train set, 4.74 (95% CI 1.02-21.93, *P* = 0.028) in the validation set, and 2.04 (95% CI: 1.00-4.17, *P* = 0.049) in the external test set. The DLR, Pathomics, and MoEMIL models successfully stratified patients into high- and low-risk groups, with Kaplan-Meier curves demonstrating statistically significant differences in OS (*P* < 0.05, Fig 4). In contrast, TNM staging failed to achieve significant risk stratification in the training and validation sets (*P* > 0.05, Fig 4). Calibration plots and decision curve analysis of models were shown in S2 Fig.

**Fig 4:**
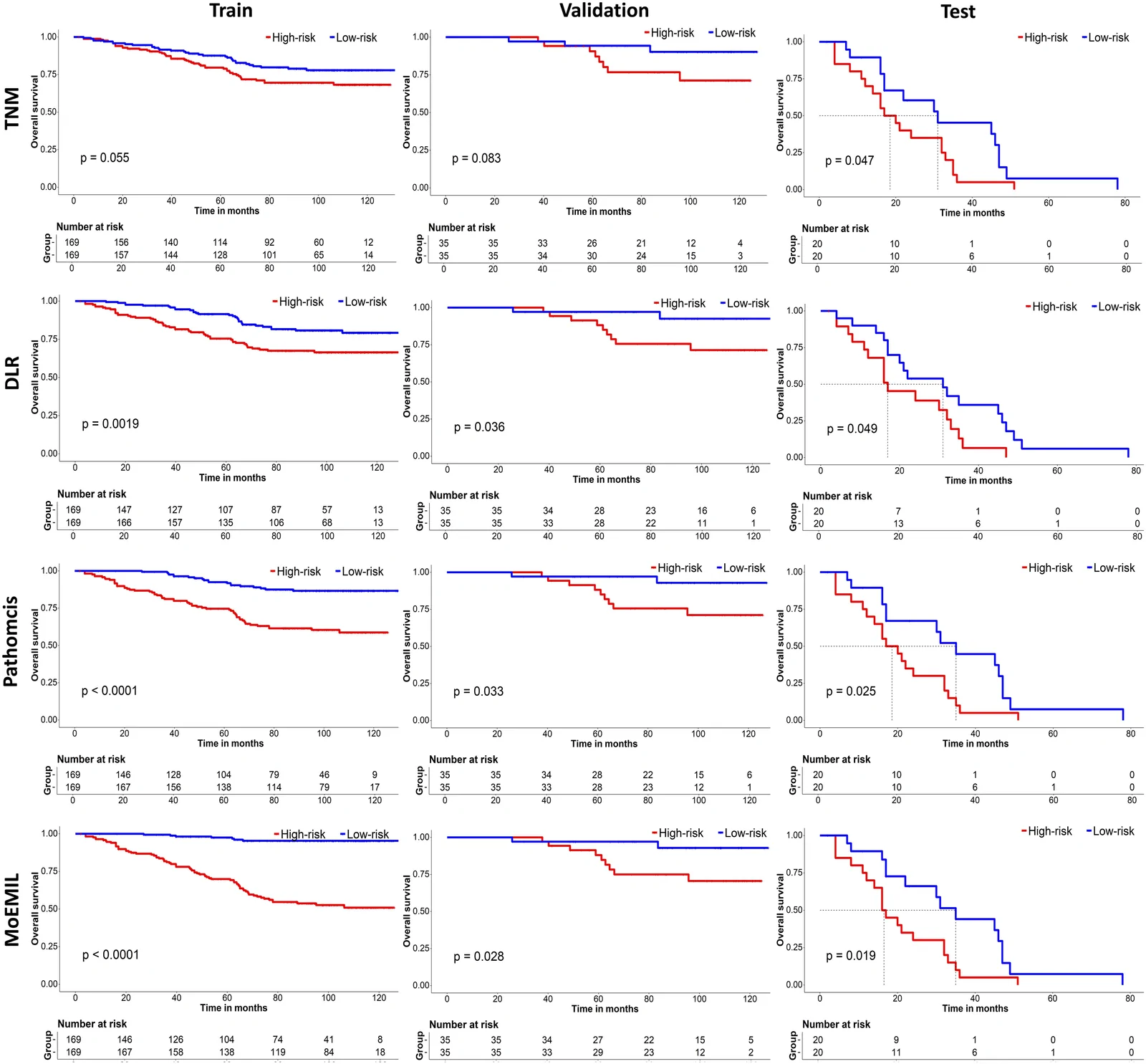
Risk stratification analysis for predicting OS in LANPC patients using TNM staging, DLR, pathomics, and MoEMIL models across both the train, validation, and test sets. Abbreviations: OS = overall survival, LANPC = locally advanced nasopharyngeal carcinoma, DLR = deep learning radiomics, MoEMIL = Mixture-of-Experts integrated Multiple Instance Learning.

### Subgroup analysis incorporating EBV-DNA and stratified by WHO type

EBV-DNA was available in 217 of 404 patients. Given the established prognostic relevance of EBV-DNA in NPC, we performed a subgroup analysis in the 217 patients (53.7% of the total cohort) with available EBV-DNA data to evaluate whether EBV-DNA provides complementary value to MoEMIL. As shown in Table C in S1 Text, within this EBV-DNA-available subgroup, the original MoEMIL model achieved an AUC of 0.747 for IC response prediction and a C-index of 0.741 for OS prediction. After incorporating EBV-DNA status as an additional input feature, the model’s performance improved to an AUC of 0.774 for IC response and a C-index of 0.760 for OS. The modest improvement with EBV-DNA addition and the high missing rate (46.3%) of EBV-DNA in the overall cohort precluded its inclusion in the primary model.

To address whether MoEMIL’s prognostic stratification is independent of histological subtype, we performed a subgroup analysis restricted to patients with WHO type III, who constituted the vast majority of the cohort. MoEMIL still significantly stratified patients into high- and low-risk groups (log-rank *P* < 0.0001; S3 Fig).

### Ablation study comparing multi-task and single-task learning models

To evaluate the contribution of the multi-task learning framework, we conducted an ablation study comparing MoEMIL against single-task versions trained with identical architecture and hyperparameters but using only the IC response head (IC-only) or only the survival head (OS-only). As summarized in Table D in S1 Text, the multi-task MoEMIL model produced marginal improvements over the single-task models, with AUC increasing from 0.777 to 0.801 for IC response and C‑index from 0.736 to 0.766 for OS.

### Interpretability and visualization analysis

For WSIs, we computed attention scores for all 256 × 256 overlapping tissue patches. These scores were then converted into percentile ranks ranging from 0.0 (low attention) to 1.0 (high attention) and spatially aligned with the corresponding WSI, averaging scores for any overlapping patches. The resulting heatmap was mapped to RGB values through a color map and overlaid on the original H&E-stained image with 0.5 transparency. This high-resolution attention heatmap highlighted the relative prognostic relevance of different image regions used for risk prediction. We observed that high-attention regions in both high- and low-risk patients corresponded with tumor tissues (Fig 5A). However, for high-risk patients, high-attention regions in WSIs predominantly corresponded to tumor cell clusters which were large syncytiform cells with unclear cell boundaries, round or oval nuclei, and large centrally located nucleoli. For low-risk patients, high-attention regions in WSIs predominantly corresponded to tumor cell clusters that were fusiform and grew in clumps, with clear boundaries and keratinized cells occasionally. For MRI, the high-risk area was also highlighted on the tumor area based on Grad-CAM (Fig 5B), which highly overlapped with the lesion area identified by the WSIs. This further validates the robustness and clinical applicability of the model.

**Fig 5:**
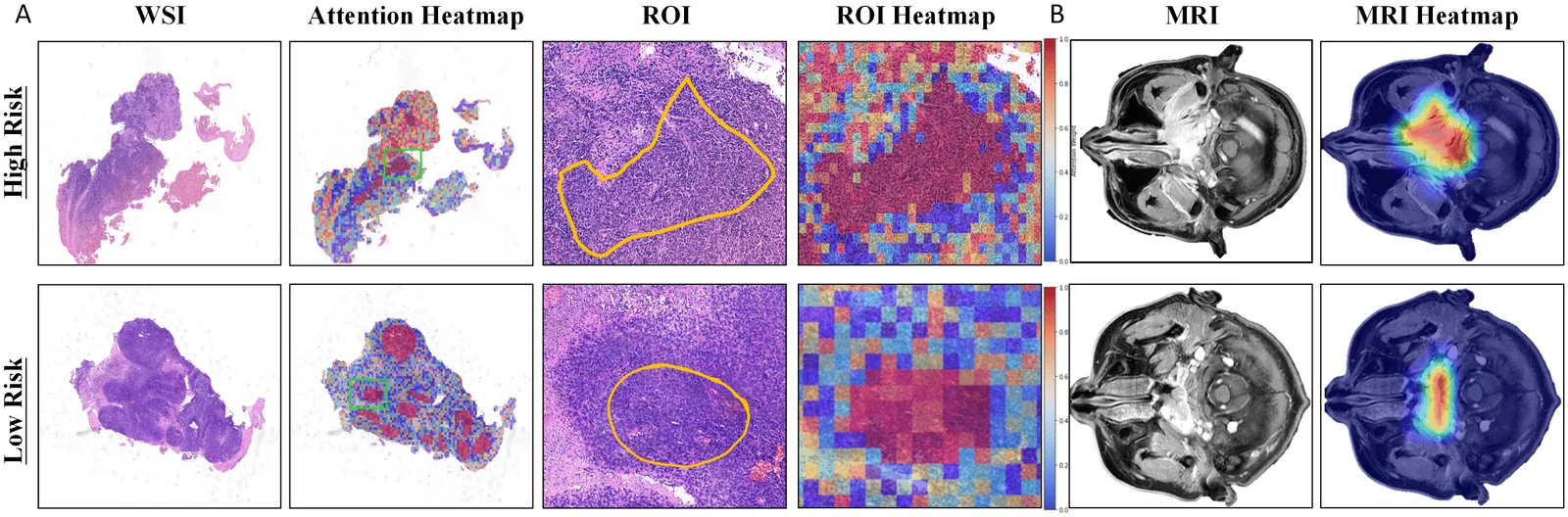
Interpretability and visualization. **(A)** Histological feature analysis: Sequential visualization from left to right includes the WSI thumbnail, attention-based risk heatmap, representative ROI, and the corresponding magnified ROI heatmap. For both high-risk (top row) and low-risk (bottom row) cohorts, the model’s attention predominantly aligns with viable tumor clusters and specific stromal microenvironments. The whole-slide attention heatmaps were generated by calculating MoEMIL-derived attention scores across overlapping patches (utilizing a 25% spatial overlap stride), providing a continuous landscape of the morphological features driving the risk prediction. **(B)** Radiological Explainability: Representative axial MRI scans displaying the primary tumor are paired with their corresponding Grad-CAM saliency maps. These heatmaps highlight the critical intra-tumoral regions that most significantly influence the model’s prognostic output. The Grad-CAM analysis confirms that the deep learning radiomics module effectively prioritizes the heterogenous core and invasive margins of the primary tumor, rather than background noise or non-specific features. Abbreviations: WSI = whole slide image, ROI = region of interest, Grad-CAM = Gradient-weighted Class Activation Mapping.

## Discussion

In this multi-center study, a novel multi-task deep learning model integrating pretreatment MRI and WSIs was developed and validated to simultaneously predict IC response and survival outcomes in patients with LANPC. The proposed MoEMIL model demonstrated superior predictive performance compared to unimodal models in both internal and external validation cohorts. To our knowledge, this is the first study to integrate MRI and WSIs within a multi-task MMoE framework for dual-endpoint prediction in LANPC, extending prior MRI-only radiomics and deep learning approaches for prognostic evaluation in NPC [33] and for IC response and survival in LANPC [13,14].

Accurate pretreatment prediction of both IC response and survival outcome in LANPC patients is crucial for developing personalized treatment plans. For IC response, MoEMIL achieved a test AUC of 0.801, outperforming TNM staging, DLR, and Pathomics models. This improvement translates into more accurate early identification of poor responders, enabling timely adjustments to CCRT, such as alternative regimens or dose intensification, while good responders can safely continue standard therapy, potentially improving outcomes and reducing toxicity. For OS, the C-index improved from 0.553 (TNM), 0.676 (DLR), and 0.690 (Pathomics) to 0.766 (MoEMIL) in the test set. High-risk patients identified by MoEMIL had a 2.04-fold higher hazard of death (95% CI: 1.00-4.17, *P* = 0.049) compared to low-risk patients. This stratification supports personalized management: high-risk patients may benefit from intensified adjuvant therapy and more frequent surveillance, while low-risk patients could be spared unnecessary treatment and follow-up intensity. However, these potential benefits remain unproven at this stage, and prospective interventional studies are required to confirm whether model-based risk stratification translates into improved patient outcomes.

Most existing models primarily depend on unimodal MRI data or integrate only clinical variables such as TNM staging and EBV-DNA status, neglecting the valuable insights that can be gained from multiscale image modalities. Radiomics captures tumor heterogeneity at the imaging level, reflecting macroscopic morphological and textural patterns, while pathomics, derived from WSIs, provides insights into cellular architecture and the tumor microenvironment. Our findings demonstrate that the DLR model relying solely on MRI data or the pathomics model relying solely on WSIs exhibits only limited to moderate performance in predicting IC response and survival outcomes. In contrast, the MoEMIL model, which integrates complementary macroscopic radiological and microscopic pathological information, substantially improves predictive efficacy. In both internal and external validation cohorts, the MoEMIL model consistently outperformed unimodal models in predicting IC response and survival outcomes. This finding aligns with the emerging paradigm of radiopathomics in other cancers [20–22,34], reinforcing the principle that multi-scale data integration enhances prognostic modeling.

Multitask learning, as opposed to learning each task in isolation, is a joint learning paradigm that harnesses the inherent correlation of multiple related tasks to achieve reciprocal benefits in improving performance, enhancing generalizability, and reducing the overall computational cost [35]. In LANPC, tumor response to IC is an established independent prognostic factor for survival after intensity-modulated radiotherapy [5]. Consequently, we hypothesized that IC response and OS share underlying biological mechanisms, such as tumor heterogeneity, cellular proliferation, and microenvironmental features, which manifest as common representations in high-dimensional feature space. By modeling these tasks jointly within a MMoE framework, our approach captures shared latent features through shared expert networks, while task-specific gating mechanisms dynamically filter information to account for the distinct nuances of short-term response versus long-term survival. This biological-to-computational synergy allows the model to learn more robust generalized features than single-task counterparts. The MoEMIL model effectively stratified patients into good- and poor-responders after IC, as well as stratified patients into high- and low-risk groups for OS. A key strength of the MoEMIL architecture lies in its dynamic feature-routing mechanism based on MMoE framework [23], where gating networks adaptively weight contributions from different experts according to each task-treatment response or survival prediction. This mechanism not only effectively utilizes shared information across multimodal data but also preserves task-specific features, thereby theoretically mitigating the common issue of negative transfer in multi-task learning and contributing to its consistent performance in external validation.

To isolate the contribution of the multi-task architecture, we performed an ablation study comparing MoEMIL against single-task versions (IC-only and OS-only) with identical multimodal inputs and hyperparameters. Multi-task learning yielded modest improvements over single-task counterparts: + 3.1% in IC response AUC and +4.1% in OS C-index. These gains are smaller than those reported in some previous multi-task learning studies for NPC [13,14]. However, direct comparisons with prior work should be made with caution. In many earlier studies, the performance advantage of multi-task models was partially attributable to the concurrent introduction of multimodal data, whereas single-task baselines often relied on unimodal inputs. By holding the multimodal input (MRI + WSI) constant across both single-task and multi-task conditions, our ablation study provides a cleaner estimate of the true value added by joint task optimization. Under this rigorous setting, the modest yet consistent improvements suggest that multi-task learning serves primarily as an effective inductive regularizer by constraining the model to learn shared representations that generalize better to unseen data, rather than as a source of large performance leaps. This regularization effect is particularly valuable given the relatively small sample size and high dimensionality of our multimodal data. We also acknowledge that the close biological relationship between IC response and OS, which reflect underlying tumor aggressiveness and treatment sensitivity, may limit the room for additional gains, as single-task models may already capture substantial shared information through the multimodal features.

Notably, the proposed MoEMIL model demonstrated superior predictive performance for both IC response and OS compared to the conventional TNM staging system. While TNM staging remains the cornerstone of clinical decision-making in LANPC, it solely relies on anatomical extent and fails to capture the underlying tumor biological heterogeneity, which limits its ability to accurately stratify patients with divergent therapeutic outcomes. In our study, TNM staging exhibited limited predictive accuracy for IC response (AUC: 0.645-0.713) and poor prognostic discrimination for OS (C-index: 0.553-0.689), with Kaplan-Meier curves failing to show significant risk stratification in the training and validation sets. In contrast, the MoEMIL model consistently achieved superior stratification across all cohorts. These findings underscore the inherent limitations of anatomical staging alone and highlight the added value of integrating multi-scale tumor characteristics through deep learning.

A further strength of this work is its emphasis on model interpretability and visualization. To elucidate the histopathological basis of predictions, we employed attention-based heatmaps that leverage learned attention scores to highlight tissue regions most influential to the model’s final outcome. In this study, high-attention regions in both high- and low-risk patients aligned with tumor areas, indicating that these regions significantly influence outcome prediction, which is consistent with our previous study [24]. In high-risk patients, these high-attention regions predominantly contained tumor cell clusters exhibiting large syncytiform cells with indistinct borders, round to oval nuclei, and prominent central nucleoli. Such morphology, as noted by Wang et al. [36], is associated with high proliferative activity, pleomorphism, and poor differentiation, features often linked to adverse prognosis. The heatmaps thus visually quantify each region’s contribution to risk prediction. Clinically, inspecting morphological details and other cellular features within high-attention areas on a WSI can aid in assessing the patient’s prognostic risk. To interpret the MRI model, Grad-CAM was applied for visualization. It calculates the gradients of the target class with respect to the final convolutional layer and uses global average pooling to produce localization maps, thereby identifying the most influential regions in the model’s decision-making process. Heatmaps intuitively display which regions of the input image are critical for the model’s prediction, helping understand the key areas the model focuses on. For instance, the WSI heatmap in Fig 5A illustrates that the model focuses on tumor cell morphology, while the MRI Grad-CAM heatmap in Fig 5B indicates the model’s attention to macroscopic tumor regions. Together, these two modalities contribute complementary information at different scales, collectively forming a comprehensive portrait of the tumor’s biological behavior. This phenomenon strongly suggests that macroscopic imaging representations and microscopic pathological morphology are not independent of each other, but rather are both rooted in the intrinsic, cross-scale biological behavior of tumors.

We observed a performance gap between the train and test cohorts, which suggests some degree of overfitting, a common challenge in deep learning studies involving relatively small sample sizes and high-dimensional multimodal data. To address this, we incorporated multiple regularization mechanisms, including L2 weight decay, dropout (rate = 0.3), early stopping, and a multi-task learning architecture that inherently reduces overfitting by enforcing shared feature representations across related tasks. Crucially, despite this gap, MoEMIL significantly outperformed the DLR model and TNM staging for both endpoints. Compared to the Pathomics model, MoEMIL achieved significant improvement for OS (*P* = 0.0049) and borderline improvement for IC response (*P* = 0.064). Its prognostic C-index and survival stratification (log-rank *P* < 0.05) remained robust in the external test set. These complementary lines of evidence suggest that while the model has not fully generalized to the level of training performance, it has captured cross-scale tumor features that are genuinely predictive. Nevertheless, prospective multi-center studies with larger, more diverse populations are essential to definitively establish the model’s generalizability and to transition it toward clinical deployment.

The current study shares a partially overlapping patient cohort with our previous work [24], in which we developed a deep learning radiopathomics model for OS prediction using a simple concatenation-based fusion of MRI and whole-slide image features. The present study extends beyond previous work in several important ways. First, while previous work was a single-task model focusing only on OS, the current MoEMIL framework simultaneously predicts both IC response and OS, enabling early treatment adaptation alongside long-term prognostic stratification. Second, we introduce a multi-gate mixture-of-experts (MMoE) architecture with task-specific gating networks that dynamically weigh shared expert representations for each endpoint. This design captures task-related commonalities while preserving task-specific features, which was not possible with the previous concatenation approach. Third, the present study incorporates enhanced interpretability tools, including CLAM heatmaps for whole-slide images and Grad-CAM for MRI, the latter of which was not implemented in previous work. Collectively, these advancements establish MoEMIL as a methodological and clinically oriented improvement over our prior work.

Our study has several limitations. First, although this was a multi-center investigation, the external validation cohort comprised only 40 patients from a single institution. Such a modest sample size may broaden the confidence intervals and reduce the precision of our performance estimates, thereby limiting the strength of our cross-center generalizability conclusions. Encouragingly, despite this limited sample, the MoEMIL model maintained robust discrimination (AUC = 0.801, C-index = 0.766) on a completely independent cohort. To further address this limitation, we are actively planning collaborative efforts with two additional high-volume medical centers to assemble larger retrospective datasets and are also designing prospective cohort studies to rigorously evaluate the model’s clinical accuracy and generalizability. Second, the external test cohort contained only 5 OS events, which limits the precision of the HR estimates and warrants cautious interpretation of the survival stratification results in this subset. Furthermore, it should be noted that no PD cases were observed in our cohort; therefore, the poor responder group consisted entirely of SD cases. Consequently, the model’s ability to distinguish SD from PD could not be assessed, and its generalizability to patients with PD remains unknown. Third, plasma EBV-DNA, an established prognostic biomarker in NPC, was available in only 217 of 404 patients. While a subgroup analysis incorporating EBV-DNA showed modest improvements in model performance, the original MoEMIL model maintained favorable performance in the same EBV-DNA-available subgroup, suggesting that the multimodal imaging and pathological features captured by MoEMIL provide substantial prognostic information independent of EBV-DNA. Nonetheless, the high missing rate (46.3%) reflects a common challenge in retrospective studies and precluded integration of EBV-DNA into the primary model without introducing potential selection bias. Future prospective studies with complete EBV-DNA collection are warranted to determine the optimal combination of MoEMIL and EBV-DNA for clinical decision support. Fourth, the vast majority of cases were WHO type II or III, whereas WHO type I cases were extremely rare (n = 1). Consequently, the model’s applicability to uncommon histological subtypes could not be meaningfully assessed. Although subgroup analysis restricted to WHO type III patients demonstrated that MoEMIL maintained significant risk stratification within this histologically homogeneous subgroup (S3 Fig), the dominant representation of undifferentiated histology limits the generalizability of our findings to other NPC subtypes. Fifth, our cohort comprised exclusively Asian patients; therefore, the model’s performance in other ethnic groups, particularly in non-endemic regions where NPC etiologies may differ, remains unknown and requires further validation. Finally, our cohort comprised exclusively adult patients (≥18 years), and no adolescents or young adults under 18 were included. Given the distinct biological and clinical characteristics of NPC in younger populations, the model’s applicability to pediatric or adolescent patients remains unknown and requires dedicated validation in future studies. Addressing these constraints in larger, prospective, multi-institutional cohorts will be essential to confirm the stability and generalizability of the MoEMIL model.

## Conclusion

In summary, we developed a novel multi-task deep learning model utilizing the MMoE framework to simultaneously predict induction chemotherapy response and overall survival by integrating radiological and pathological data. MoEMIL shows potential as an adjunctive tool in risk stratification and treatment planning, but prospective multi-center validation is necessary.

## Supporting information

S1 Text Supplementary tables. (DOC)

S1 Fig Calibration (A-C) and decision curve analysis (D-F) for IC response among the TNM, DLR, Pathomics, and MoEMIL models in train, validation and test sets. Abbreviations: IC = induction chemotherapy, DLR = deep learning radiomics,MoEMIL = Mixture-of-Experts integrated Multiple Instance Learning. (TIF)

S2 Fig Time-dependent calibration plots at 5 years (A-C) and decision curve analysis (D-F) for OS among the TNM, DLR, Pathomics, and MoEMIL models in train, validation and test sets. Abbreviations: OS = over survival, DLR = deep learning radiomics, MoEMIL = Mixture-of-Experts integrated Multiple Instance Learning. (TIF)

S3 Fig Subgroup Analysis Stratified by WHO Type. Abbreviations: WHO = world health organization. (TIF)
