# Supplementary material for: Multi-task deep learning integrating pretreatment MRI and whole slide images predicts induction chemotherapy response and survival in locally advanced nasopharyngeal carcinoma
Source: PLOS Digit Health. 2026 Oct 5;5(10):e0001378. doi: 10.1371/journal.pdig.0001378 (PMC13637888; doi:10.1371/journal.pdig.0001378)
Supplement: Supplementary file 1 [file pdig.0001378.s001.doc]

**Supplementary Tables**

**Table A** Cohort overlap between the current study and prior work [24]

| Cohort | Current study (n) | Overlap with [24] (n) | Newly added (n) | Excluded from [24] (n) | Reason for exclusion |
| --- | --- | --- | --- | --- | --- |
| Primary cohort (Hunan Cancer Hospital) | 364 | 293 | 71 | — | — |
| External cohort (First Affiliated Hospital of Jinan University) | 40 | 40 | 0 | 10 | Incomplete IC response assessment |
| Total | 404 | 333 | 71 | 10 | — |

**Table B DeLong tests for AUC comparisons and paired bootstrap for C-index comparisons between models in the external test set**

| Comparison (Test Set) | Metric | MoEMIL | Comparator | *P*-value |
| --- | --- | --- | --- | --- |
| MoEMIL vs. Pathomics | AUC (IC response) | 0.801 | 0.752 | 0.064 |
| MoEMIL vs. DLR | AUC (IC response) | 0.801 | 0.709 | 0.015 |
| MoEMIL vs. TNM | AUC (IC response) | 0.801 | 0.645 | <0.001 |
| MoEMIL vs. Pathomics | C-index (OS) | 0.766 | 0.690 | 0.0049 |
| MoEMIL vs. DLR | C-index (OS) | 0.766 | 0.676 | <0.001 |
| MoEMIL vs. TNM | C-index (OS) | 0.766 | 0.553 | <0.001 |

**Table C The predictive and prognostic performance of two models in EBV-DNA-available subgroup**

| Model | IC Response | | OS |
| --- | --- | --- | --- |
|  | AUC | ACC | C-index |
| MoEMIL | 0.747 | 0.790 | 0.741 |
| EBV-DNA-integrated MoEMIL model | 0.774 | 0.816 | 0.760 |

**Table D Ablation study comparing multi-task and single-task learning models**

| Model | IC Response | | OS |
| --- | --- | --- | --- |
|  | AUC | ACC | C-index |
| IC-only | 0.777 | 0.775 | — |
| OS-only | — | — | 0.736 |
| MoEMIL (Multi-task) | 0.801 | 0.797 | 0.766 |
